# Matrix Metalloproteinase 11 Promotes Migration and Invasion of Colorectal Cancer by Elevating Slug Protein

**DOI:** 10.7150/ijms.98007

**Published:** 2024-08-13

**Authors:** Chaomin Pan, Jingping Dai, Yiyi Wei, Li Yang, Zhuoyu Ding, Xinke Wang, Juan He

**Affiliations:** 1 Guangdong Provincial Key Laboratory of Gastroenterology, Department of Gastroenterology, Nanfang Hospital, Southern Medical University, Guangzhou, China.; 2 Department of Gastroenterology, Longgang Central Hospital of Shenzhen, Shenzhen, Guangdong, China.

**Keywords:** colorectal cancer, MMP11, metastasis, Slug, tumor microenvironment

## Abstract

**Purpose:** Matrix metalloproteinase-11 (MMP11), which belongs to the stromelysin subgroup, has been reported to play a role in the progression of colorectal cancer (CRC). However, the significance of MMP11 in the tumor microenvironment, immune/stromal cells, and its mechanism in CRC remain unclear.

**Methods:** The impact of MMP11 knockdown using specific short hairpin RNAs (shRNAs) on the metastasis and invasion of colorectal cancer RKO and SW480 cells was investigated using western blot, quantitative real-time polymerase chain reaction (qRT-PCR), transwell assays, and immunohistochemistry.

**Results:** MMP11 mRNA expression was significantly higher in CRC cells than in normal cells, and its expression was stimulated in CCD-18Co fibroblasts. Additionally, MMP11 expression was found to be higher in individuals aged ≤ 65 years, the T4/T3 group, and Stage III/IV patients. Overall survival (OS) and disease-free survival rates were significantly different between the high and low MMP11 groups. Furthermore, the receiver operating characteristic (ROC) curves for MMP11 at 1-, 3-, and 5-years were 0.450, 0.552, and 0.560, respectively. Moreover, MMP11 promoted the migration and invasion of CRC cells by elevating the expression of Slug protein. Most importantly, MMP11 was positively associated with M0-macrophages and negatively associated with M1-macrophages, NK cells activated, NK cells resting, T cells CD4 memory activated, and T cells follicular helper, indicating the remarkable interactions of MMP11 with tumor immunology.

**Conclusions:** MMP11 plays an important role in colorectal cancer development, and its mechanism in CRC needs to be further explored in the future.

## Introduction

CRC accounts for almost 10% of all cancers and is the second leading cause of cancer-related deaths, with 1.9 million new cases and 0.9 million cancer-related deaths globally by 2020 1, 2. One-fifth of those diagnosed with CRC will develop metastatic CRC 3, and 40% will experience a recurrence after prior localized treatment 4. The prognosis of metastatic CRC is poor, with less than 20% of patients surviving for five years 3. Despite advancements in CRC treatment, the overall survival rate has not improved significantly due to recurrence and metastasis. Therefore, researchers are exploring potential molecular markers to better understand the progression of CRC metastasis and develop future treatment strategies to reduce its occurrence.

Matrix metalloproteinases (MMPs) possess the ability to modify the tumor microenvironment (TME), and their expression and activation are heightened in nearly all human cancers when contrasted with normal tissues 5. MMPs are proteolytic enzymes that break down proteins and regulate various cellular behaviors, including cancer cell growth, differentiation, migration, and invasion, as well as immune surveillance 5. It has been shown that stromal MMPs, such as MMP-9, which are particularly produced by infiltrating inflammatory cells, exhibit anti-tumorigenic properties 6. MMP-7, a matrilysin protein, degrades collagen, proteoglycans, elastin, laminin, fibronectin, and casein. This could suggest a more aggressive phenotype of colon cancer 7. MMP11 distinguishes itself from other MMPs by not being secreted as a pro-MMP and instead being activated immediately upon secretion through intracellular activation by furin within the constitutive secretory pathway of the trans-Golgi network 8. Due to its unique structure, MMP11 plays a distinct role in tumor development compared to other MMPs. MMP11 can be released by tumor cells in an autocrine manner, which actively modifies the TME's interaction with it to adjust it to its biologically malignant behaviors 9. However, the prevalence of MMP11 in the TME remains unclear, highlighting the need for further research into the precise functions of MMP11.

MMP11, also referred to as stromelysin-3, is a member of the MMP family and was initially identified in breast cancer 10. Previous studies have suggested that MMP11 regulates cancer cell proliferation, tumor migration, invasion, and metastasis in various cancers 11, 12. It has been proposed that MMP11 is highly expressed in colonic carcinomas 13. Additionally, individuals with colon cancer who have higher blood levels and mRNA expression of MMP11 have a worse prognosis 14, 15. MMP11 is believed to have an extremely complex function and is involved in various signaling pathways, such as JAK/STAT, TGF-β, MAPK, Wnt, and PI3-kinase, and/or MMPs inducers 12, 16. Su *et al.* found that IGF-1 induced MMP11 expression may promote the proliferation and invasion of gastric cancer SGC-7901 cells through the JAK/STAT3 pathway 17. Ying Zhang *et al.* used enrichment analysis on data from breast cancer samples to determine that MMP11 may have a downstream target in the TGF-β signaling pathway 18. However, the mechanism of action of MMP11 in CRC remains unclear.

In this investigation, the expression level of MMP11 was examined in CRC and adjacent normal tissues using clinical samples. The relationship between MMP11 and clinical characteristics, as well as its impact on prognosis, was explored using bioinformatics analysis. Furthermore, transwell assays were employed to confirm the role of MMP11 in CRC. Bioinformatic analyses such as functional enrichment analysis, immune infiltration, and immunotherapy were utilized to investigate the molecular mechanism of MMP11 in CRC. These findings enhance our understanding of MMP11 in CRC and contribute to the development of novel, accurate, and practical CRC detection methods.

## Materials and methods

### Data collection and preprocessing

**Figure 1:**
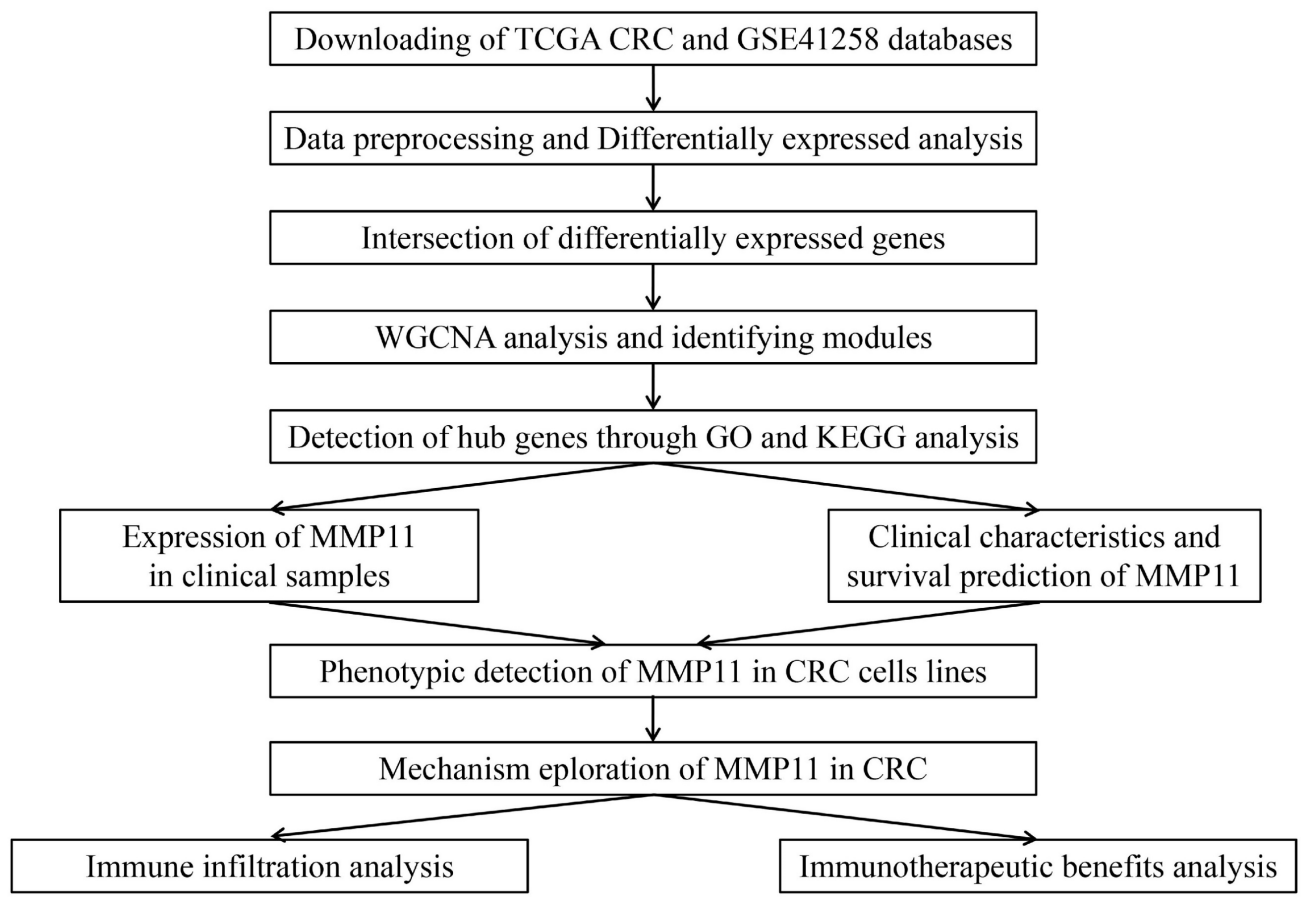
The flowchart to summarize the analysis procedure of this study.

A flowchart of this study was depicted in Figure 1. Gene expression and clinical characteristics of CRC and adjacent normal tissues were retrospectively obtained from publicly accessible datasets sourced from the Cancer Genome Atlas (TCGA) database (portal.gdc.cancer.gov) and the Gene Expression Omnibus (GEO) database (www.ncbi.nlm.nih.gov/geo). For the TCGA and GSE41258 processing, the probe ID of the gene expression microarrays was matched to the gene symbol using log2 logarithmic transformation and quantile normalization. The average values of genes with numerous probes were computed as gene expression levels, which were fulfilled using the Affy package of R software 19. The limma package in R software was employed to detect genes that were abnormally expressed 20. The differentially expressed genes (DEGs) between TCGA CRC samples and GSE41258 were intersected.

### Construct the WGCNA and do a functional enrichment analysis

Weighted gene co-expression network analysis (WGCNA) is a widely used algorithm that is based on high-throughput gene co-expression profiling to identify co-expression networks in various diseases 21. To construct a scale-free co-expression network of all genes, we utilized the WGCNA package in R 22. We then clustered the genes using the average-linkage hierarchical clustering method and set the minimum gene size for each module at 50. The function “pickSoftThreshold” was employed to determine the soft-thresholding power β. To quantify the degree of network connectivity, we converted adjacency into a topological overlap measure (TOM). Modules were represented by branches of the hierarchical clustering dendrogram based on TOM dissimilarity. We utilized the “blockwiseModules” function to construct the gene network in a single step 23.

To further investigate the modules, the dissimilarity of module eigengenes (ME) was assessed, and similar modules were combined. The cutoff criterion for combining the modules was set at 0.25, and modules with similarity values greater than 0.75 were included. To determine the correlation between clinical features, MMP11, and modules, Pearson's correlation test was used. The gene significance (GS) and module membership (MM) were also calculated. Intersections of genes in the selected modules were conducted in the TCGA CRC and GSE41258 databases, and the hub genes were subjected to Gene Ontology (GO) and Kyoto Encyclopedia of Genes and Genomes (KEGG) enrichment analyses.

### Patients and clinical samples

A total of 44 pairs of cancerous and normal tissue samples were collected from patients who underwent surgical treatment at Nanfang Hospital of Southern Medical University after obtaining their informed consent. Each patient sample was given a histological diagnosis of colorectal cancer. The cancerous and normal tissue samples were stored at -80 ℃ until use. The Protection of Human Subjects Committee at Nanfang Hospital of Southern Medical University approved the protocols used in this study (IRB approval number: NFEC-2013-098, approval date: December 18, 2013). Informed consent was obtained from the donors in writing to participate in research involving human tissue samples.

### Cell culture and transfection

The human normal colon epithelial cell line (NCM460), as well as human CRC cell lines (HCT116, RKO, SW480, SW620, and LoVo), and human intestinal CCD-18Co fibroblast cell lines, were obtained from the Cell Bank of Type Culture Collection (CBTCC, Chinese Academy of Sciences, Shanghai, China). These cells were cultured in Dulbecco's modified Eagle medium (DMEM) (Gibco, Carlsbad, CA), which was supplemented with 10% fetal bovine serum (FBS; Gibco, Carlsbad, CA). The cells were maintained in an environment that was rich in water and contained 5% CO2.

The siRNAs for MMP11 (si-MMP11) and the negative control (NC) were synthesized by GenePharm (Shanghai, China). The siRNA sequence for MMP11 was as follows: MMP11 sense, 5'-GTGCTGACATCATGATCGA-3'. Cells (2 × 10^5^ per well) were seeded in 6-well plates 24 hours before transfection using Lipofectamin^TM^ 3000 (Invitrogen, UAS), following the manufacturer's protocol.

The lentivirus carrying the shRNA against MMP11 (Lv-sh) and a control vector without any shRNA (Lv-NC) were generated using the designed shRNAs (OBiO, China). These shRNAs were transfected into RKO and SW480 cells. The sequence of the shRNA used was 5'-GTGCTGACATCATGATCGA-3'. The cells were seeded at a density of 2.0 × 10^5^ cells per well in 6-well plates 24 hours before transfection. Transfection was conducted using lentiviral particles (RKO multiplicity of infection (MOI) = 10; SW480 MOI = 10) and polybrene (5 ug/ml), according to the manufacturer's protocol. After 24 hours, the medium containing the virus was replaced with a complete medium. Seven days after transfection, puromycin (Solarbio, China) was added to all cells at a final concentration of 5 ug/ml for the selection of resistant colonies. These colonies were then isolated and further studied.

### RNA isolation and qRT-PCR

Total RNA was isolated from the cells or tissues using Trizol solution (TaKaRa, China). qRT-PCR was conducted using the PrimeScript RT Reagent Kit and SYBR Premix Ex Taq (TaKaRa, Dalian, China), following the manufacturer's instructions. The expression of glyraldehyde-3-phosphate dehydrogenase (GAPDH) was utilized as a reference for our results. The primers used were listed in Table S1. The 2^-△△Ct^ technique was applied to the qRT-PCR results to determine the Ct values of the amplified products.

### Cell proliferation and transwell assays *in vitro*

Cell proliferation was carried out using a Cell Counting Kit-8 (CCK-8) (ApexBIO, Japan). RKO and SW480 knockdown cells were seeded in 96-well plates and cultivated for 0, 24, 48, 72, and 96 hours, respectively. Following this, 10 ul of CCK-8 was added to each well and incubated for 2 hours. The absorbance value (OD) at 450 nm was measured using a microplate reader.

Cell migration and invasion assays were carried out using transwell chambers (8.0 μm pore size, Corning). The lower chamber was filled with 600 μl of 10% FBS medium, and then transfected knockdown RKO and SW480 cells (2.5 × 10^5^) in 200 μl of serum-free media were carefully inserted into each filter insert (upper chamber). The cells were incubated at 37 ℃ for 48 hours, followed by the removal of the filter inserts from the chambers, fixation in methanol for 15 minutes, and staining with hematoxylin for 15 minutes. The samples were cleaned, dried, and placed on slides. After staining with blue, the number of migrating cells was counted in five different fields under an inverted microscope for statistical analysis. The invasion assays were conducted in a similar manner to the migration assays, with the exception of placing the cells on a Matrigel-coated membrane (LYNJUNE Matrix, China), as previously described 24. The number of invading cells was quantified from five random fields at 100× magnification.

### Immunohistochemistry staining

For the purpose of conducting immunohistochemistry (IHC) analysis, CRC samples were treated with 10% formaldehyde and embedded in paraffin. Subsequently, 4-μm-thick sections of continuous paraffin were prepared. Following the manufacturer's guidelines, deparaffinization and antigen retrieval were performed. Next, the sections were exposed to anti-MMP11 (T55778S, 1:1000, Abcam) antibodies. Following this, secondary antibodies (PV-6001, ZSGB-BIO) were applied, and the sections were visualized using a DAB chromogenic agent (ZLI-9017, ZSGB-BIO). Finally, the sections were examined under a microscope.

### Western blot analysis

All proteins were extracted from cells using RIPA lysis buffer, which was combined with a loading buffer. The extracted proteins were then mixed and separated by sodium dodecyl sulfate-polyacrylamide gel electrophoresis (10% SDS-PAGE) before being transferred to polyvinylidene difluoride (PVDF) membranes. The membranes were blocked with a 5% non-fat milk solution for an hour and then incubated with primary antibodies (anti-GAPDH, E-cadherin, N-cadherin, Vimentin, Snail, 1:1000, Proteintech) (Slug, MMP11, 1:1000, Abcam) according to the manufacturer's instructions. GAPDH was utilized as the reference in the process, and the membranes were washed three times with TBST before being incubated with secondary antibodies for an hour at room temperature. An ECL chemiluminescene system was implemented to detect the signal, while ImageJ software was employed to evaluate the relative protein expression.

### Bioinformatic analysis

Gene Set Variation Analysis (GSEA) was employed to investigate the potential biological processes of MMP11 in CRC using TCGA. GSEA was conducted using the R package “clusterprofiler” 25. The tumor microenvironment conditions were evaluated quantitatively by calculating the levels of stromal and immune cell infiltration using expression profiles obtained from the TCGA dataset. The R packages “ESTIMATE” 26 and “CIBERSORT” 27 were used to estimate the immune score, stromal score, and 22 types of immune cell infiltration. The Wilcoxon t-test was employed for the calculation of each score to compare between the high and low MMP11 groups.

The measure of tumor mutation burden (TMB) reflects the number of somatic mutations per coding area of a genome. It has been suggested that heavily mutated tumors can produce a substantial number of neoantigens, leading to an increase in T-cell infiltration and potentially enhanced responsiveness to checkpoint blockade 28. Pearson's correlation test was performed to identify the correlation between TMB and MMP11 expression levels.

The immunogenicity of a tumor is influenced by various genes related to effector cells, immunosuppressive cells, major histocompatibility complex (MHC) molecules, and immune regulatory factors. Using bioinformatics analysis, immunogenicity can be assessed and quantified. Immunophenoscores (IPS) of patients with colon and rectal cancers were obtained from the Cancer Imaging Archive (TCIA) database (https://tcia.at/) 29, 30. To predict the sensitivity of immunotherapy, we compared the IPS between the high and low MMP11 groups for various immunotherapy decisions.

### Statistical analysis

The data were analyzed using the R (version 4.2.2) and R Bioconductor packages. Quantitative data were displayed as the mean ± standard deviation derived from a minimum of three replicates. A significant level of *P* < 0.05 was used in all two-sided statistical analyses, and figures were generated using GraphPad Prism 7.0 (GraphPad Software, Inc.). One-way analysis of variance (ANOVA) was used for multiple group comparisons, and *P < 0.05, **P < 0.01, and ***P < 0.001 were considered statistically significant. Survival curves for prognostic analysis were constructed using the Kaplan-Meier method, and log-rank tests were employed to evaluate differences between groups. A univariate Cox regression model was used to determine the hazard ratio (HR) of MMP11. A multivariate Cox regression model was used to confirm whether MMP11 was an independent predictor. Additionally, a conventional nomogram with a calibration curve was created using the “rms” R package.

## Results

### Differentially expressed genes (DEGs) identification

**Figure 2:**
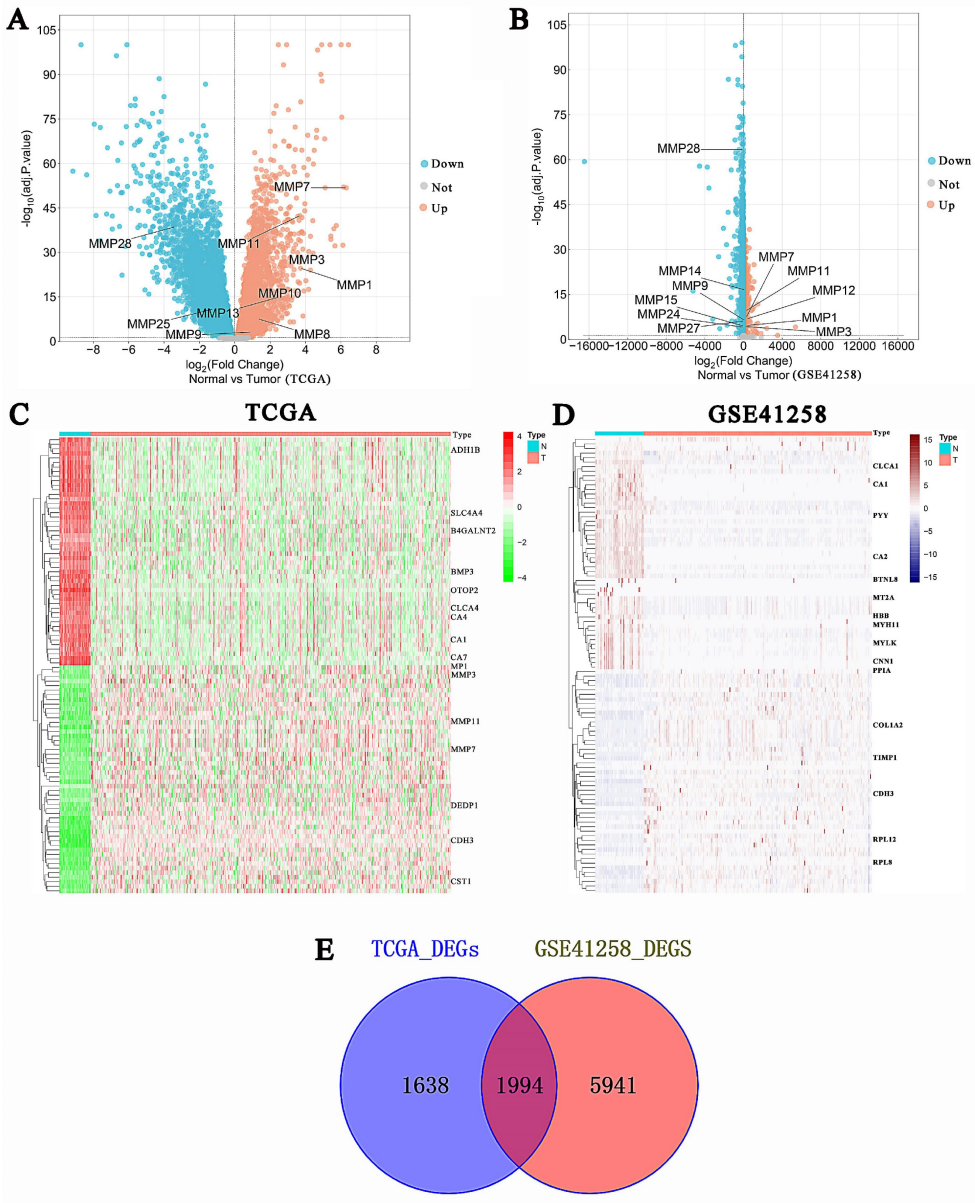
Identification of CRC DEGs. (A-B) Volcano plots of DEGs between tumor and normal tissues in the TCGA CRC and GSE41258 databases. In the volcano plots, red dots indicated upregulated genes, while blue dots represented downregulated genes. (C-D) The heatmaps demonstrated the expression levels of DEGs. Green and navy blue indicated a low expression, while red and dark red indicated a high expression. (E) The Venn diagram showed the overlap of DEGs in the TCGA CRC and GSE41258 databases.

The TCGA database was used to investigate the function of mRNAs in CRC, including 488 tumor samples and 42 normal samples. Another database was GSE41258, containing 54 normal colon samples, 47 polyps, 186 primary tumors, 47 liver metastases, 20 lung metastases, and 12 cell lines. Furthermore, we divided the GSE41258 dataset into two groups: 54 normal colon samples and 253 tumor samples (186 primary tumors, 47 liver metastases, and 20 lung metastases). DEG identification was performed, which finally obtained 3,632 DEGs in the TCGA CRC database and 7,935 DEGs in the GSE41258 database. The volcano plots of DEGs were displayed in Fig. 2A-B, in which we only depicted genes of the MMPs family. In the TCGA volcano plot, MMP-1, MMP-3, MMP-7, MMP-8, MMP-10, and MMP-11 were upregulated, whereas MMP-28 and MMP-25 were downregulated. Similarly, MMP-1, MMP-3, MMP-7, MMP-11, MMP-12, MMP-14, and MMP-24 were upregulated in the GSE41258 volcano plot, whereas MMP-15, MMP-27, and MMP-28 were downregulated. Moreover, MMP-9 was not significant in TCGA and GEO differentially expressed analyses. We also identified genes of the MMP family in the TCGA heatmap (Fig. 2C), including MMP-1, MMP-3, and MMP-11. However, there was a significant variation in the gene expression of the GSE41258 database, and we only exhibited the top 17 most expressed genes across the GSE41258 different samples (Fig. 2D). The DEGs between the two databases were then intersected, and 1,994 intersection genes were obtained for follow-up analysis (Fig. 2E).

### WGCNA construction and hub-genes detection

**Figure 3:**
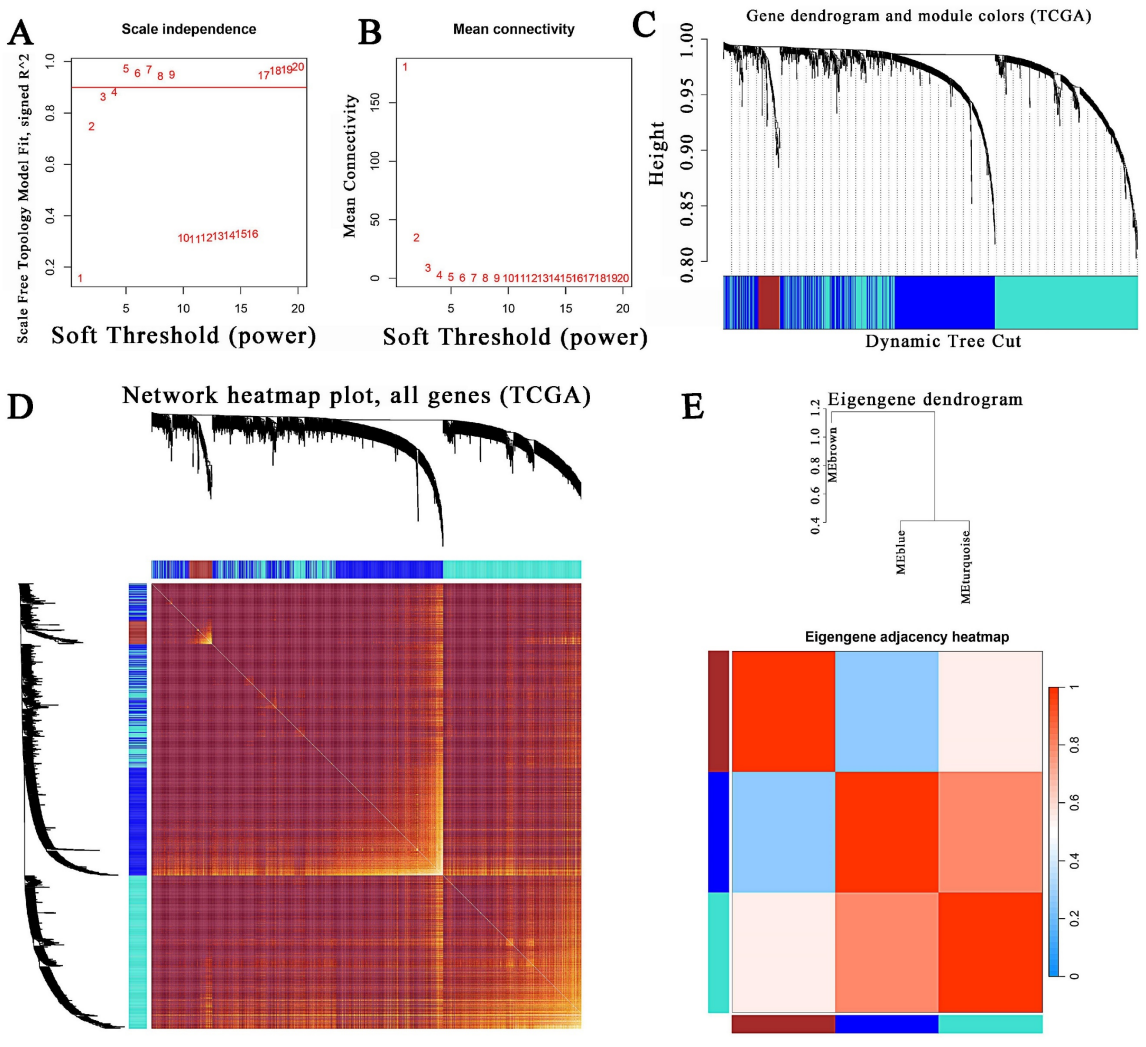
Determination of soft-thresholding power and construction of co-expression modules in the TCGA CRC database. (A) Scale-free fit index analysis for different soft-thresholding powers (β). (B) Mean connectivity analysis of various soft-thresholding powers. (C) The gene clustering dendrogram was created via hierarchical clustering of the TOM-based dissimilarity. The colored row below the dendrogram signifies the module colors. (D) A heatmap visualization of the topological overlap matrix of 1000 randomly chosen genes. Single genes were represented by rows and columns, and as the colors became darker, they indicated lower topological overlap, while increasing lightness indicated higher topological overlap. (E) Hierarchical clustering dendrogram and heatmap of the module eigengenes. Colors represented the intensity of adjacency. TOM, topological overlap measure.

The “WGCNA” package in R 4.2.2 was performed to determine the most cooperative gene modules between normal and tumor tissues in the 1,994 intersection genes of the two databases. There were no outliers in sample clustering (Figure 3). With a cutoffR^2^ value of 0.9, the soft threshold β in TCGA was 3 (Figure 3A-B). Subsequently, hierarchical clustering analysis based on weighted correlation was performed, and the clustering results were divided into groups according to the established criteria for creating gene modules. Three modules were identified in the gene co-expression network. Among them, the maximum and minimum modules comprised 730 genes (turquoise) and 74 genes (brown). The gray module contained one gene that did not belong to any other module and was omitted. The gene hierarchical clustering dendrogram, along with the assigned module colors, was presented in Figure 3C. The topological overlap matrix was shown by a heatmap supplementary to hierarchical clustering dendrograms and modules (Fig. 3D). Dendrogram clusters and heatmaps of ME from each module were plotted to illustrate the similarity of each module (Fig. 3E). The results for the GSE41258 database corresponding to this section were displayed in Figure S1.

**Figure 4:**
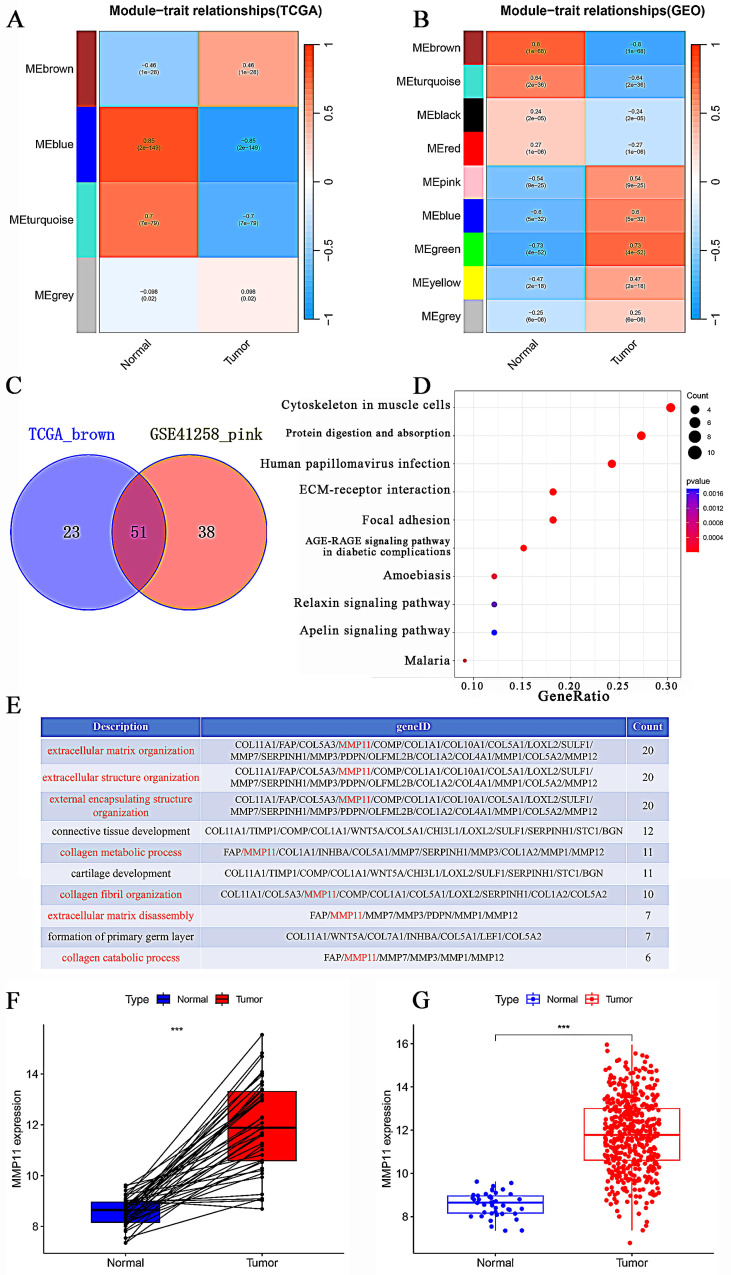
WGCNA construction and hub-gene identification. (A-B) Module-trait relationships between modules and clinical features in the TCGA CRC and GSE41258 databases. Each row represented a gene module, and each column represented a clinical feature. (C) Venn diagram of the TCGA MEbrown and GSE41258 MEpink modules. (D) KEGG enrichment analysis of 51 hub genes. (E) The top 10 biological processes identified in the GO analysis. (F) Differences in MMP11 expression between tumor and normal groups in the TCGA CRC database. (G) Differential expression of MMP11 in CRC and paraneoplastic tissues in the TCGA CRC database. *p < 0.05, **p < 0.01, ***p < 0.001.

The module-trait correlations between modules and phenotypes were displayed in Figures 4A-B. The TCGA MEbrown module had the strongest correlation with the tumor (*P* = 1e-28, cor = 0.46). The GSE41258 MEgreen also exhibited the highest correlation with the tumor (*P* = 4e-52, cor = 0.73), followed by MEblue (*P* = 5e-32, cor = 0.60), and MEpink (*P* = 9e-25, cor = 0.54). Genes between TCGA MEbrown and GSE41258 MEgreen were intersected. Nevertheless, no intersection genes were identified. Next, we intersected genes between TCGA MEbrown and GSE41258 MEblue, which identified only five genes. These five genes were OSM, IFI6, CXCL10, and S100A2. Following a thorough literature search, we discovered that these five genes had been positively or negatively linked to CRC. Ultimately, we decided to intersect the TCGA MEbrown and GSE41258 MEpink modules to detect hub genes. Ultimately, 51 hub genes were identified (Fig. 4C).

51 hub-genes were analyzed using KEGG analysis, and the results revealed that these pathways were related to protein digestion and absorption, ECM-receptor interaction, and focal adhesion. (Fig. 4D). The extracellular matrix was linked to seven of the top ten biological processes (BP) sequenced by gene count in GO analysis, whereas formation and development were linked to the remaining three BP (Fig. 4E). Ultimately, we identified MMP11 as an intersecting gene in these seven BPs. In the TCGA database, MMP11 mRNA was shown to be substantially higher in CRC tissues than in the adjacent normal tissues (Fig. 4F). Matched tissues also displayed a substantial variation in MMP11 expression (Fig. 4G).

### Clinical characteristics and prognostic prediction of MMP11

**Figure 5:**
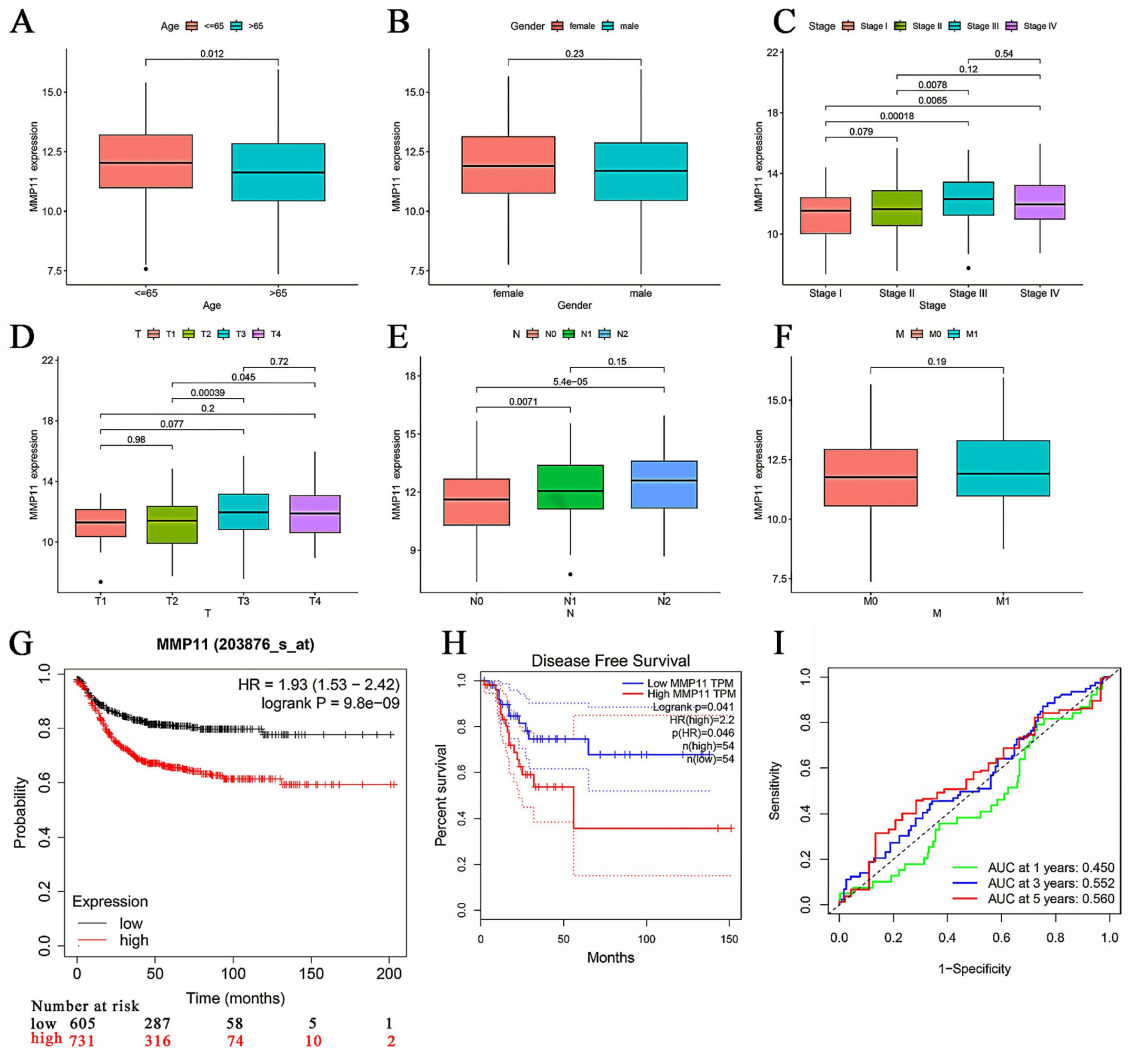
Clinical characteristics of MMP11 and its prognostic value. (A-F) MMP11 expression in CRC samples and normal tissues of different ages, genders, stages, and TNM classifications. (G) Kaplan-Meier analysis of CRC patients with high or low expression of MMP11, as determined by data from the TCGA CRC database. (H) Disease-free survival rate in CRC patients with high or low expression of MMP11, data from GEPIA. (I) ROC curve of MMP11 in the TCGA CRC database. *p < 0.05, **p < 0.01, ***p < 0.001. TNM, tumor node metastasis; ROC, receiver operating characteristic; GEPIA, Gene Expression Profiling Interactive Analysis.

To further investigate the connection between MMP11 and CRC, we examined the clinical characteristics obtained from the TCGA database. We discovered that MMP11 expression was strongly correlated with age, tumor node metastasis (TNM) classification, and stage. MMP11 was highly expressed in people aged ≤ 65 years (Fig. 5A). MMP11 expression did not significantly correlate with the characteristics of gender (Fig. 5B).

MMP11 expression was significantly higher in stage III/IV tumors than in stage I/II (Fig. 5C). In particular, MMP11 was more highly expressed in the T4/T3 group than in the T2/T1 group (Fig. 5D). CRC samples with lymph node invasion tended to have higher MMP11 expression than those without lymph node invasion (Fig. 5E). However, this trend was not observed in the characteristic of distant metastasis (Fig. 5F), owing to the small sample sizes. We used Gene Expression Profiling Interactive Analysis (GEPIA), which contained TCGA and GTEx CRC samples, to explore the survival prediction of MMP11. The overall survival (OS) rate of MMP11 was significantly different from that of low- and high-risk patients (hazard ratio (*HR*) = 1.93,* P* = 9.8e-09) (Fig. 5G). There was a significant difference in the disease-free survival rate between the low- and high-risk groups (*HR* = 2.2,* P* = 0.041) (Fig. 5H). Additionally, the receiver operating characteristic (ROC) curves for MMP11 at 1-, 3-, and 5-years were 0.450, 0.552, and 0.560 (Fig. 5I). A nomogram including all the prognosis-related correlated parameters (i.e., age, gender, TNM classification, and stage) was created to better estimate the prognosis of CRC patients (Figure S2A). The nomogram accuracy in predicting the 1-, 3-, and 5-year survival rates was demonstrated by the calibration curves (Figure S2B). These results suggested that MMP11 may play an important role and may be a potential prognostic marker for colorectal cancer.

### Expression of MMP11 in clinical samples

**Figure 6:**
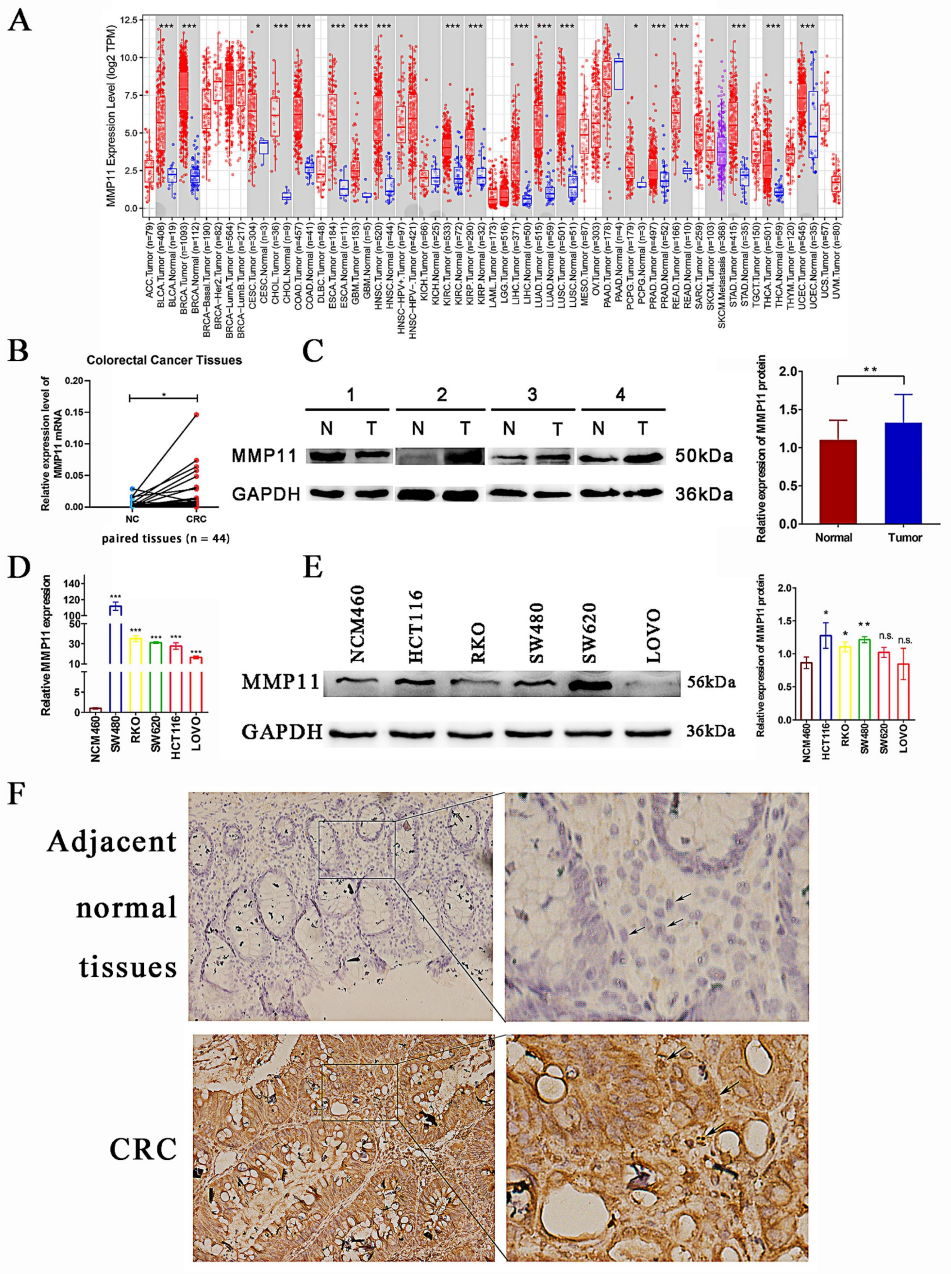
Differential expression of MMP11. (A) Differential expression of MMP11 in different tumor and normal tissues obtained from the TIMER database. (B-C) The expression levels of MMP11 in CRC and normal tissues were determined using qRT-PCR and western blot assays. The qRT-PCR experiments were repeated three times for each of the 44 tissue pairs. The western blot assays were repeated once for each of the 32 paired tissues. (D-E) The expression levels of MMP11 in colonic mucosal epithelial cells (NCM460) and CRC cell lines (SW480, RKO, SW620, HCT116, and LoVo) were determined using qRT-PCR and western blot assays. The qRT-PCR and western blot assays were repeated three times. (F-G) Immunohistochemistry was performed to assess the expression of MMP11 in CRC samples and adjacent normal tissues, which was repeated three times. The black arrow indicated the location of MMP11. GAPDH was used as a control to normalize the expression of MMP11. The data were presented as the mean ± SD, and the experimental results were statistically analyzed using Student's t-test, and the significance levels were indicated in the figure. TIMER, tumor immune estimation resource; MMP, matrix metalloproteinase; CRC, colorectal cancer; qRT-PCR, quantitative real-time polymerase chain reaction. n.s. *p* > 0.05, *p < 0.05, **p < 0.01, ***p < 0.001, data was shown as the mean ± SD.

The above results connected CRC's low survival rate with MMP11's high expression. To confirm these findings, analyses were performed on clinical CRC samples. As shown in Figure 6A, MMP11 mRNA expression was significantly higher in most cancers than in corresponding normal tissues, including colon and rectal cancers. In terms of RNA and protein levels, the results were similar in matched colorectal tissues (Fig. 6B-C). The expression of MMP11 was high in CRC cell lines, particularly in HCT116, SW480, and RKO cells at the RNA and protein levels (Fig. 6D-E). Additionally, immunohistochemical analysis of CRC samples and adjacent normal tissues confirmed that MMP11 was highly expressed in tumor tissues (Fig. 6F). These results highlighted the high expression of MMP11 and revealed its importance in CRC.

### Knockdown of MMP11 impacted migration and invasion of CRC cells *in vitro*

**Figure 7:**
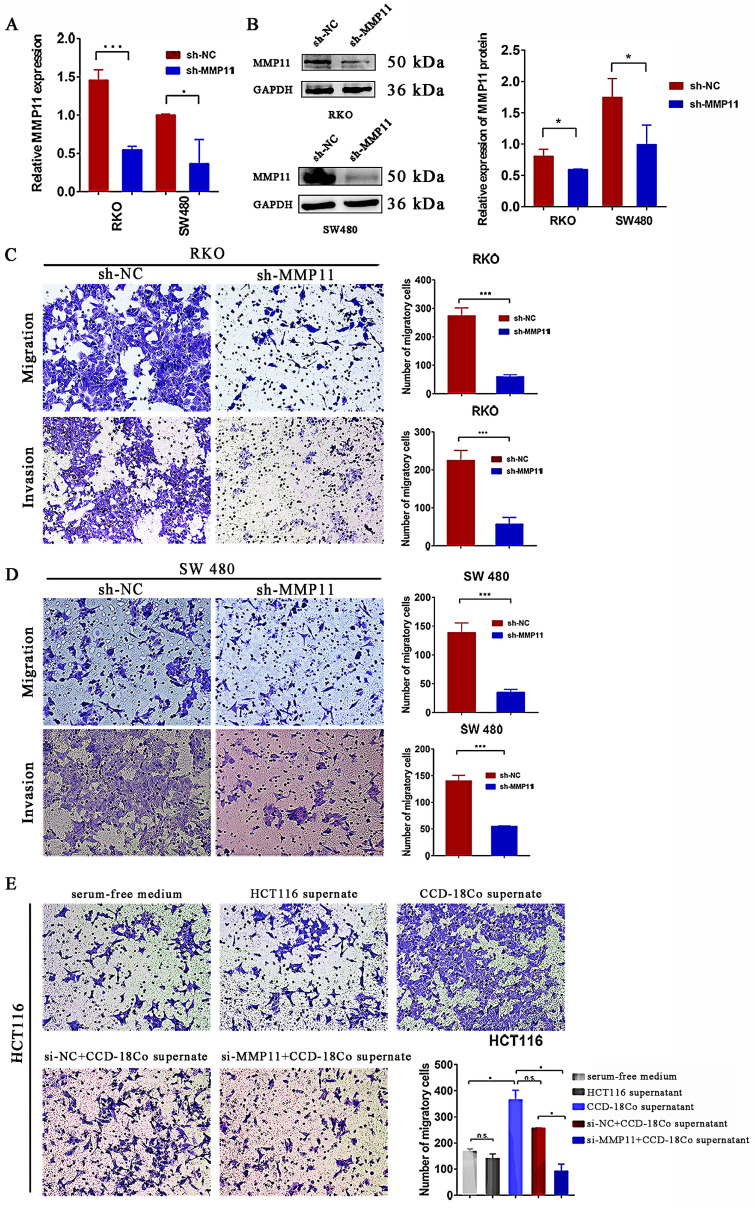
Impact of MMP11 on migration and invasion of CRC cells. (A-B) qRT-PCR and western blotting revealed the expression of MMP11 in RKO and SW480 cell lines transfected with shRNA. The qRT-PCR and western blot assays were repeated three times. (C-D) Transwell assays of RKO and SW480 cells. The cells were stained with crystal violet. The transwell assays were repeated three times in RKO and SW480 cells. (E) siRNA transfection of HCT116 cells for 48 hours resulted in reduced migration at the protein level. The transwell assay for siRNA in HCT116 cells was repeated three times. The experimental results were statistically analyzed using the Student's t-test, and the significance levels were indicated in the figure. The results were statistically significant, as indicated by the p values. *p < 0.05, **p < 0.01, ***p < 0.001, compared to the negative control (NC) group.

To explore whether MMP11 had an impact on the biological behavior of CRC cells, transwell assays were performed. MMP11 Lv-shRNA was used to investigate whether MMP11 knockdown inhibited the migration and invasion of CRC cells. Lv-shRNA significantly reduced the expression of MMP11 mRNA and protein (Fig. 7A-B). MMP11 knockdown markedly inhibited migration and invasion of RKO cells (Fig. 7C) and SW480 cells (Fig. 7D). To investigate the relationship between MMP11 and fibroblast cells, we separated the CCD-18Co cell supernatant and co-cultured it with HCT116 cells. According to the results, groups that received CCD-18Co cell supernatant exhibited higher HCT116 cell migration than groups that did not receive it or those that received serum-free medium (Fig. 7E). Furthermore, we co-cultured HCT116 si-MMP11 and HCT116 si-NC cells with the supernatant from CCD-18Co cells. Compared to the control groups, the results showed that the migration of HCT116 si-MMP11 cells in the groups containing CCD-18Co cell supernatant was impaired, suggesting that MMP11 may be induced by CCD-18Co cells and MMP11 may increase the migration of CRC cells (Fig. 7E). Nevertheless, MMP11 knockdown did not inhibit the progression of RKO and SW480 cells (Figure S3).

### Knockdown of MMP11 impaired the expression of Slug protein

**Figure 8:**
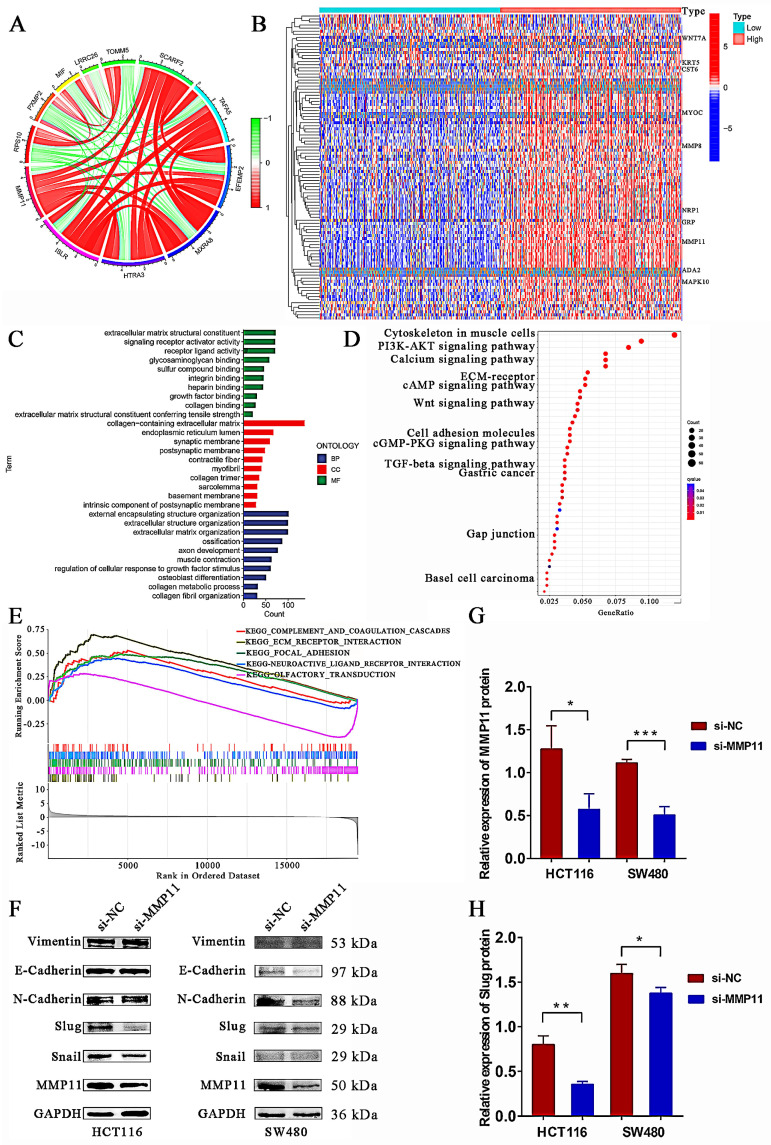
Mechanistic exploration of MMP11 expression in CRC. (A) The coexpression circle map of MMP11 in the TCGA database. (B) Heatmap of DEGs in differential MMP11 expression groups based on the TCGA database. (C-D) Enriched GO and KEGG pathways were explored in high- and low-MMP11 groups. (E) GSEA enrichment analysis was used to detect the functions and pathways of MMP11. (F) Western blot analysis of EMT markers (Vimentin, E-cadherin, N-cadherin, Slug, and Snail) after treatment with siRNA for 48 hours; GAPDH was used as an internal reference. The western blot assays for EMT markers were repeated three times. (G-H) The relative expression of MMP11 and Slug protein in si-NC and si-MMP11. NC, negative control. *p < 0.05, **p < 0.01, ***p < 0.001. The data was shown as the mean ± SD and was analyzed by a Student's t-test.

Co-expression analysis is a useful tool for examining gene interactions with target genes. We extracted five genes with the highest and five genes with the lowest co-expression coefficient of MMP11 to create the co-expression circle map in the TCGA database (Fig. 8A). TCGA tumor samples were divided into two groups according to the median expression of MMP11. DEG analysis was used to detect significant gene expression in the high and low MMP11 groups. The heatmap was displayed in Fig. 8B, which only displayed the top 10 differentially expressed gene symbols. A total of 1,173 DEGs were detected in the high and low expressions of MMP11 in the TCGA database. Subsequently, it was discovered that enriched GO terms were especially pertinent to the collagen-containing extracellular matrix (Fig. 8C). For enriched KEGG pathways, the pathways entitled “PI3K-AKT signaling pathway,” “TGF-β signaling pathway,” and “Wnt signaling pathway” were found to be directly correlated with MMP11 (Fig. 8D). GSEA was performed using the R package “clusterprofiler.” We employed the GSEA enrichment method based on gene sets from KEGG to analyze the high and low MMP11 groups (Fig. 6E). Subsequently, we detected the protein levels of epithelial-to-mesenchymal transition (EMT) markers (Fig. 8F). The results depicted that MMP11 knockdown decreased the expression of Slug, while MMP11 knockdown had no significant effect on the expression of vimentin, epithelial cadherin (E-cadherin), neural cadherin (N-cadherin), and Snail (Fig. 8F-H).

### Expression of MMP11 associated with immune infiltration of CRC

**Figure 9:**
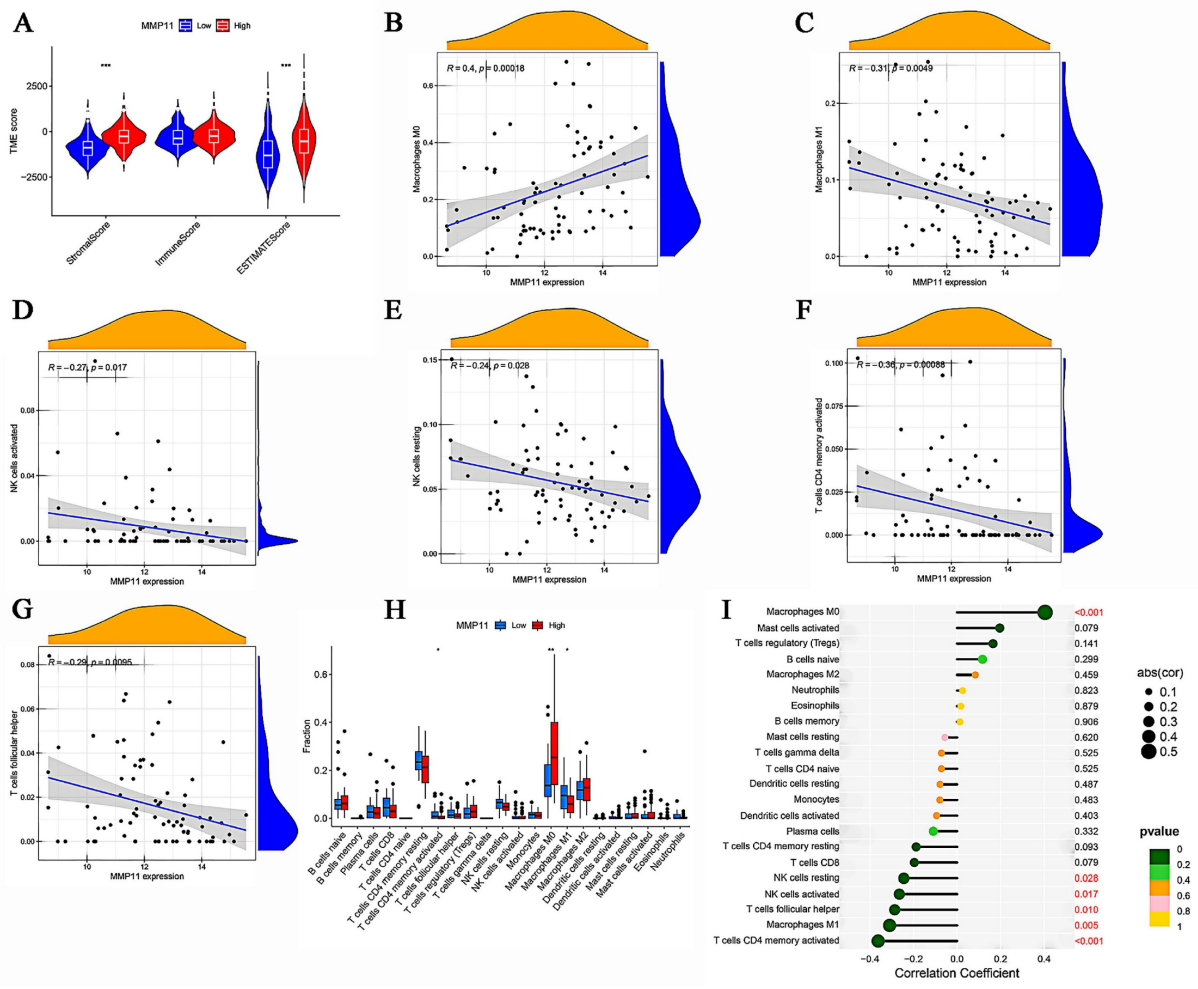
Immune microenvironment analysis. (A) A vioplot of the TME score for high and low expression of MMP11. (B-G) Pearson's correlation analysis revealed the relationship between MMP11 expression and immune cells. (H) Variations in immune cell infiltration levels between high and low MMP11 expression. (I) Lollipop was drawn to reveal the correlation between MMP11 expression and immune features. TME, tumor microenvironment.

It is commonly accepted that cancers are fundamentally dynamic ecosystems in which subclone populations of most cancer and non-malignant cells in the tumor microenvironment collaborate to advance the disease 31. Consequently, it was necessary to examine the overall look of the tumor microenvironment, and the R package “ESTIMATE” was employed for this purpose. With the exception of the immune score, the stromal and ESTIMATE scores were found to be statistically significant, and higher scores were observed in MMP11 tumor tissues (Fig. 9A). Next, we analyzed the correlation between MMP11 expression and stromal, immune, and ESTMATE scores. MMP11 expression was positively correlated with Macrophages M0 (Fig. 9B) and was negatively correlated with Macrophages M1, NK cells activated, NK cells resting, T cells CD4 memory activated, and T cells follicular helper (Fig. 9C-G). Similar differences with statistical significance existed between the high and low MMP11 groups, demonstrating the remarkable interactions between MMP11 and tumor immunology (Fig. 9H-I).

### MMP11 had the potential to predict the immunotherapeutic benefits

**Figure 10:**
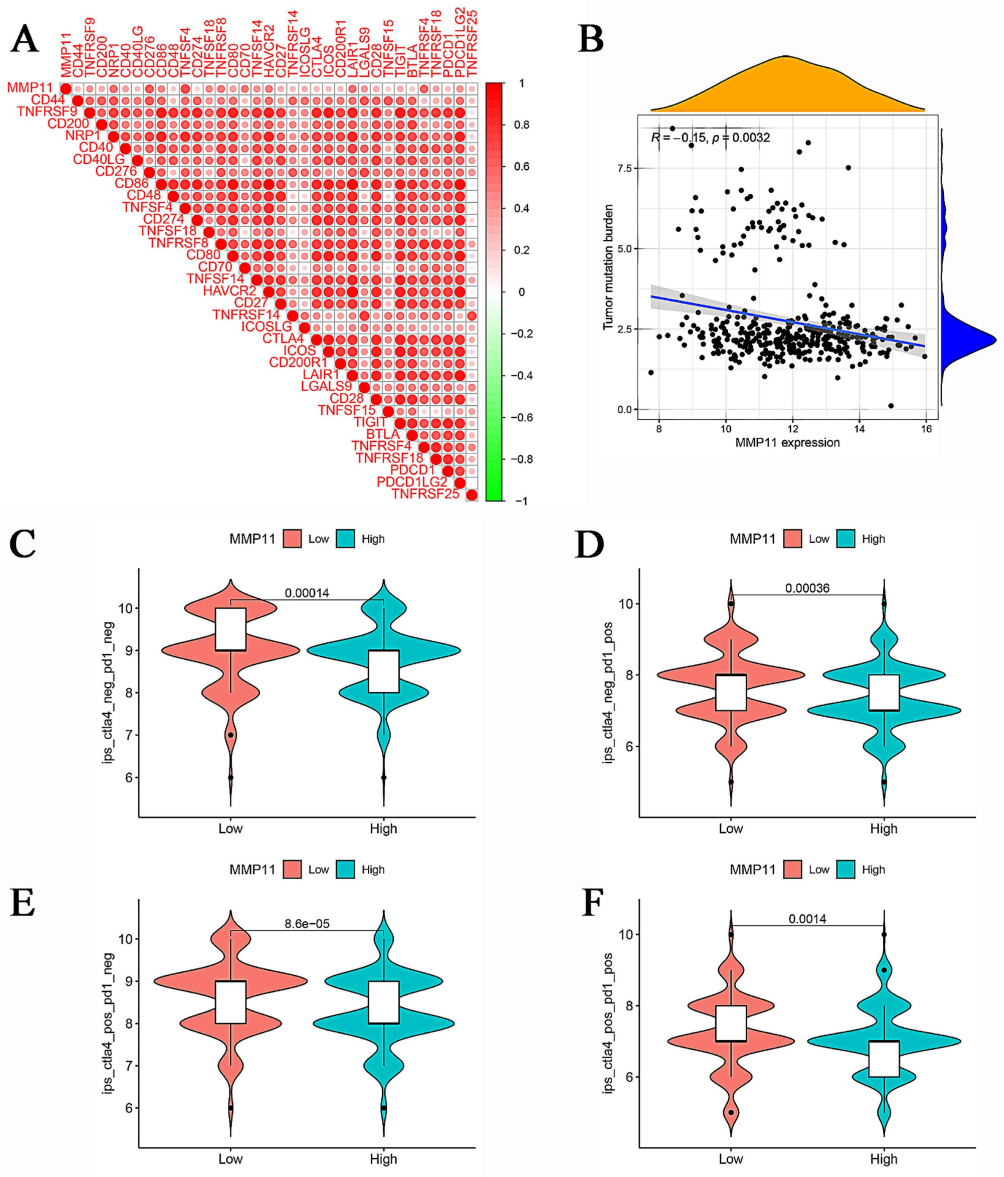
Correlation between MMP11, TMB, and IPS. (A) The relationship between MMP11 and immune checkpoint genes. (B) Relationship between tumor mutation burden and MMP11 expression. (C-F) The relationship between IPS and different MMP11 expression groups in the TCGA database. TMB, tumor mutation burden; IPS, immunophenoscore. The statistical differences were compared using the Wilcoxon test.

The aforementioned results demonstrated the relationship between MMP11 and macrophages, NK cells, and T cells. A gene cooperation analysis was conducted in CRC to explore the connection between MMP11 expression and immune checkpoint-related genes. These findings revealed that MMP11 expression was correlated with most immunosuppressive genes (Fig. 10A).

Moreover, we analyzed the relationship between the tumor mutation burden and MMP11 expression. MMP11 expression was adversely correlated with the tumor mutation burden, as shown in Figure 10B. Furthermore, we used the Cancer Imaging Archive (TCIA) to confirm MMP11 expression for the prediction of immunotherapeutic benefits. TCIA presents the results of extensive immunogenomic investigations using next-generation sequencing data for 20 solid cancers obtained from TCGA and other sources. Ips_ctla4_neg_pd1_neg, ips_ctla4_neg_pd1_pos, ips_ctla4_pos_pd1_neg, and ips_ctla4_pos_pd1_pos were significantly different between high and low MMP11 expression groups. All things considered, MMP11 may be a useful predictor of CRC patients' immunotherapeutic outcomes (Fig. 10C-F).

## Discussion

This study demonstrated a strong correlation between MMP11 expression and clinical characteristics such as age, TNM classification, and CRC stage. In addition, the expression levels of MMP11 can be utilized to identify patients at a higher risk of cancer recurrence. For instance, Cheng *et al.* discovered a correlation between MMP11 overexpression and patients with poorly differentiated tumors (*P* (MMP11) = 0.01) and lymph node metastases (*P* (MMP11) = 0.004) 32. Lucie *et al.* reported a significant correlation (*P* = 0.0073) between increased MMP11 expression and lymph node involvement. This suggested that MMP11 expression could serve as a valuable independent prognostic biomarker with potential clinical implications 33. MMP11 expression has also been found to have predictive value in gastric carcinoma, indicating its role in predicting outcomes and monitoring recurrence during follow-up 34. Patients with higher MMP11 expression had a noticeably shorter overall survival period than those with lower expression 35. These findings implied that MMP11 might be a promising biomarker for predicting the prognosis of CRC patients.

Next, we explored the role of MMP11 in CRC. MMP11 expression was up-regulated in CRC tissues, and this led to increased migration and invasion of CRC cells. However, the proliferation of CRC cell lines was not impacted by si-MMP11 in our study. A previous study by Kwon *et al.* 36 elucidated that the upregulation of MMP11 increased the migration and invasion of breast cancer cells. Porte *et al.* 37 also revealed a connection between high MMP11 transcript expression and the development of local invasion and liver metastasis in CRC. Additionally, several MMPs interacted with one another or depended on other MMPs for activation. For example, the combined signal of MMP11 and MMP19, which both cleave aggrecan and gelatin, resulted in a larger area under the curve (AUC) value than individually in thyroid cancer 38. ProMMP-2 could be activated by MMP-1, -13, -14, -15, and -16, whereas proMMP-9 could be activated by MMP-2, -3, -7, and -13 39. These results implied that MMPs proved to be more accurate predictors when examined together than when considered individually. Furthermore, MMP-2, MMP-7, and MMP-9, in conjunction with trypsin, appeared to play a special role in the proliferation of CRC 40. Several MMPs promoted the proliferation of CRC cells. MMP-1, for instance, could stimulate cell proliferation in CRC through the EMT and Akt signaling pathways, potentially compensating for the limited functions of other MMP members 41. However, there is no direct evidence demonstrating that the interaction of MMP11 with other MMPs affects the proliferation of CRC. Therefore, it remains unclear which MMPs compensated for MMP11 in promoting the proliferation of CRC.

Tumor cells can release MMP11 in an autocrine manner, which significantly impacts the tumor microenvironment and interacts with it to promote tumors' malignant growth 9. To further explore the underlying mechanisms by which MMP11 promoted the migration and invasion of CRC, bioinformatics analysis was conducted, which revealed that differential MMP11 expression was correlated with the “PI3K-AKT signaling pathway,” the “TGF-β signaling pathway,” and the “Wnt signaling pathway.” The results demonstrated that MMP11 knockdown reduced the expression of Slug but did not affect the expression of Vimentin, E-cadherin, N-cadherin, or Snail. Slug is a key transcription factor in the EMT process that binds specifically to a subset of E-box motifs in target promoters, such as the E-cadherin promoter 42, 43. Slug is overexpressed in colorectal cancer 44. Elevated levels of Slug are associated with decreased E-cadherin 45. While it was previously believed that EMT regulators function in a redundant manner, recent studies suggest that Slug has unique functions. Additionally, Slug is regulated by ubiquitination and degradation 46. Slug has the ability to bind wild-type p53 and murine double minute 2 (MDM2) simultaneously 46. In non-small-cell lung cancer, wild-type p53 upregulates the MDM2-ubiqutin ligase, leading to the formation of a p53-MDM2-Slug complex. This complex facilitates MDM2-mediated Slug degradation and inhibits cancer cell invasion. The N-terminal region of Slug is responsible for binding to both p53 and MDM2. Lin *et al.* demonstrated that the STAT3/Slug axis regulated EMT-like phenotypes in invasive glioblastoma multiforme (GBM) cells 47. Activation of STAT3 was found to promote the motility and invasion of GBM cells. Tanno *et al.* demonstrated that c-Myb transcriptionally induces Slug expression through Myb-binding sites in the Slug gene's promoter region and the first intron 48. Slug is known to mediate the effect of oncogene c-Myb on migration and invasion of cancer cells. Based on these, we hypothesized that increased MMP11 expression in CRC may stimulate the expression of Slug, which promotes the migration and invasion of CRC through a unique pathway. Further research is necessary to fully understand the mechanism by which MMP11 binds to Slug and promotes the migration and invasion of CRC cells.

MMP11 has also been reported to degrade several molecular targets, including insulin-like growth factor binding protein-1, laminin receptors, and the native alpha-3 chain of collagen VI 49. In the basement membrane, collagen VI interacted with collagen IV to enhance cell adhesion, and the reduction of collagen VI can facilitate the transition from adipocytes to fibroblast-like phenotypes. Overexpression of MMP11 was involved in the cleavage of the α3 chain of collagen VI, resulting in the accumulation of cancer-associated fibroblasts that contributed to extracellular matrix (ECM) stiffness and degradation 50, 51. Moreover, MMP11 may activate several signaling pathways, such as ERK/MAPK, growth factor-1 (IGF1) / protein kinase B (AKT) / forkhead box protein O1 (FoxO1), and others, to regulate cell invasion and metastasis 49. However, further research is necessary to fully understand how MMP11 directly promotes the migration and invasion of the CRC.

The tumor microenvironment has a significant impact on carcinogenesis by modifying epithelial cells and their capacity to develop malignant tumors 52, 53. To investigate the differences in immune cell infiltration abundance between the MMP1 high- and low-expression subgroups, we analyzed the data from an immune cell infiltration perspective. MMP11, a secreted protein, plays an important role in the microenvironment by affecting both the tumors and the ECM. Our findings suggested that MMP11 was positively correlated with macrophages M0 and negatively related to macrophages M1, NK cells activated, NK cells resting, T cells CD4 memory activated, and T cells follicular helper, indicating the remarkable interactions between MMP11 and tumor immunology. Macrophages M0 were a subset of undifferentiated macrophages that have the potential to differentiate into M1 or M2 macrophage subtypes 54. Research on cancer stem cell characterization in CRC revealed that an abundance of macrophage M0 existed in immunosuppressive subtypes and was associated with high stemness risk 55. Besides, Xu X *et al.* reported that macrophages M0 were significantly higher in hepatocellular carcinoma tissues, and high infiltration of M0 macrophages was linked to poor overall survival 56. Moreover, macrophages M0 enrichment was found to correlate with pro-tumor transcriptomic biomarkers and gene set abundance 57. Based on these studies, macrophages M0 played a critical role in immunosuppression and tumor progression. Macrophages M1, on the other hand, produced interleukin-6 (IL-6), interleukin-23 (IL-23), reactive oxygen species (ROS), and other inflammatory mediators to enhance the inflammatory response, which impeded the growth of cancer 58. According to various strategies, natural killer (NK) cells can eliminate cancer cells 59. Elizabeth L. McMichael used flow cytometry to reveal that activated NK cells produced cytokines and chemokines with anticancer effects and recruited macrophages and T cells to inflammatory sites 60. NK cells have been known to promote tumor immune escape and exhibit reduced invasion. Consequently, we hypothesized that MMP11 enabled tumor cell immune escape in CRC by modifying the immune microenvironment.

MMP11 served as a promising target for immunotherapy since it was primarily expressed in the majority of primary solid cancer tissues and metastatic lesions. Several studies have shown that MMP11 has therapeutic potential. Peruzzi *et al.* 61 demonstrated that vaccination with MMP11 can disrupt immune tolerance and offer antitumor protection in a colon adenocarcinoma mouse model. Furthermore, lung adenocarcinoma tumor development has been reported to be inhibited by MMP11 antibody treatment.^9^ In our study, we utilized the Cancer Immunome Atlas to validate MMP11 expression and predict the advantages of immunotherapy. We found that ips_ctla4_neg_pd1_neg, ips_ctla4_neg_pd1_pos, ips_ctla4_pos_pd1_neg, and ips_ctla4_pos_pd1_pos were significantly different between high and low expressions of MMP11. Overall, targeting MMP11 may potentially be an efficient strategy for slowing the progression of CRC.

In conclusion, our present results revealed a strong correlation between MMP11 expression and age, TNM classification, and CRC stage. MMP11 was found to be upregulated in CRC tissues, and it promoted CRC cell migration and invasion. Mechanistically, MMP11's cancer-promoting role in CRC was linked to the expression of Slug. Additionally, MMP11 expression levels were correlated with immune cell infiltration, making it a potential target for immunotherapy. Thus, MMP11 plays an important role in colorectal cancer development, and its mechanism in CRC needs to be further explored in the future.

## Supplementary Material

Supplementary figures and table.
