# Supplementary material for: Matrix Metalloproteinase 11 Promotes Migration and Invasion of Colorectal Cancer by Elevating Slug Protein
Source: Int J Med Sci. 2024 Aug 13;21(11):2170–88. doi: 10.7150/ijms.98007 (PMC11373555; doi:10.7150/ijms.98007)
Supplement: Supplementary file 1 — Supplementary figures and table. [file ijmsv21p2170s1.pdf]

Table S1. List of qRT-PCR primers.

Gene	Forward primer (5' - 3')	Reverse primer (5' - 3')
MMP11	CCGCAACCGACAGAAGAGG	ATCGCTCCATACCTTTAGGGC
GAPDH	CGAGCCACATCGCTCAGACA	GTGGTGAAGACGCCAGTGGA

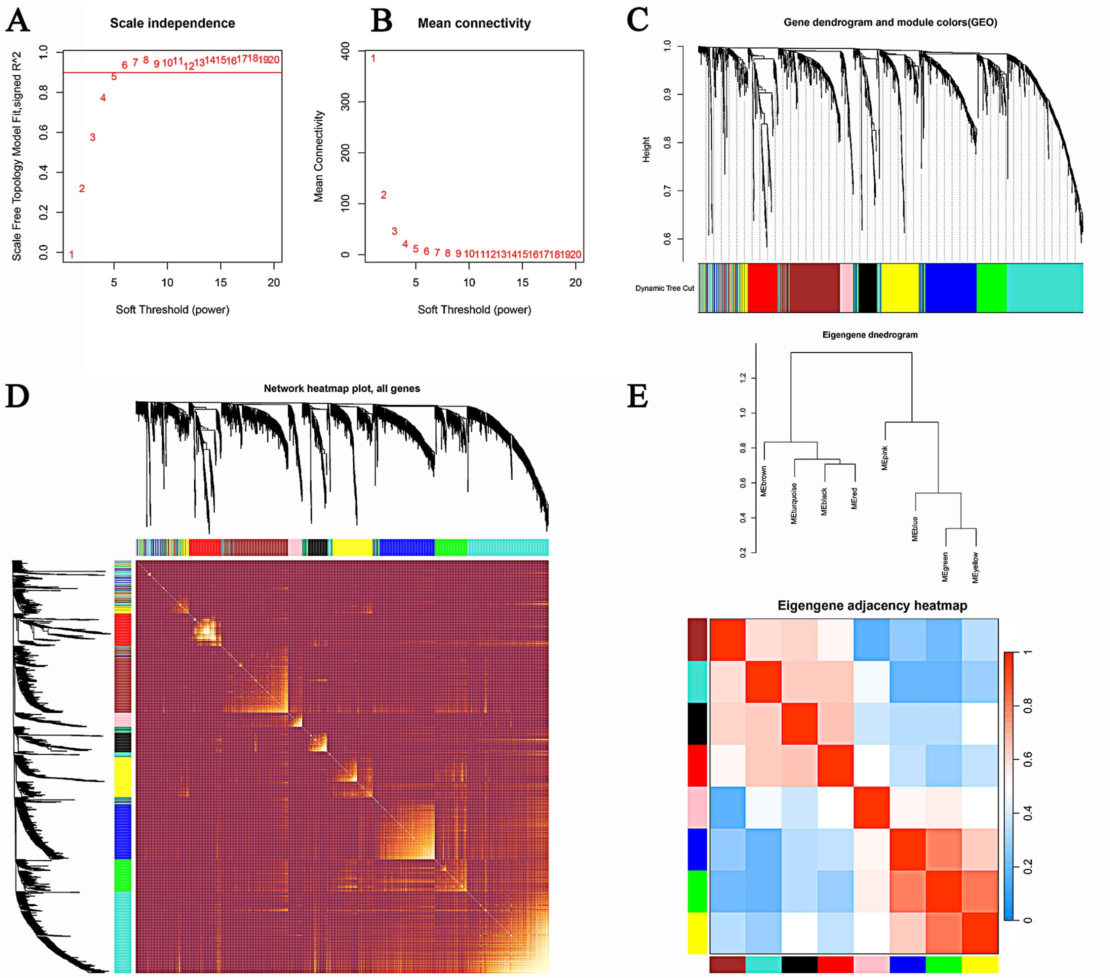

Figure S1. Evaluation of soft-thresholding power and construction of co-expression modules in the TCGA CRC database. (A) Analysis of scale-free fit index for various soft-thresholding powers (β). (B) Assessment of mean connectivity analysis for different soft-thresholding powers. (C) Creation of a gene clustering dendrogram via hierarchical clustering of TOM-based dissimilarity. The color row below the dendrogram indicated module colors. (D) Visualization of topological overlap matrix among 1000 randomly chosen genes using a heatmap. Genes were represented by rows and columns, and color intensity indicated the level of topological overlap, with darker colors indicating lower topological overlap and lighter colors indicating higher topological overlap. (E) Hierarchical clustering dendrogram and heatmap of module eigengenes. Colors represented the intensity of adjacency. TOM, topological overlap measure.

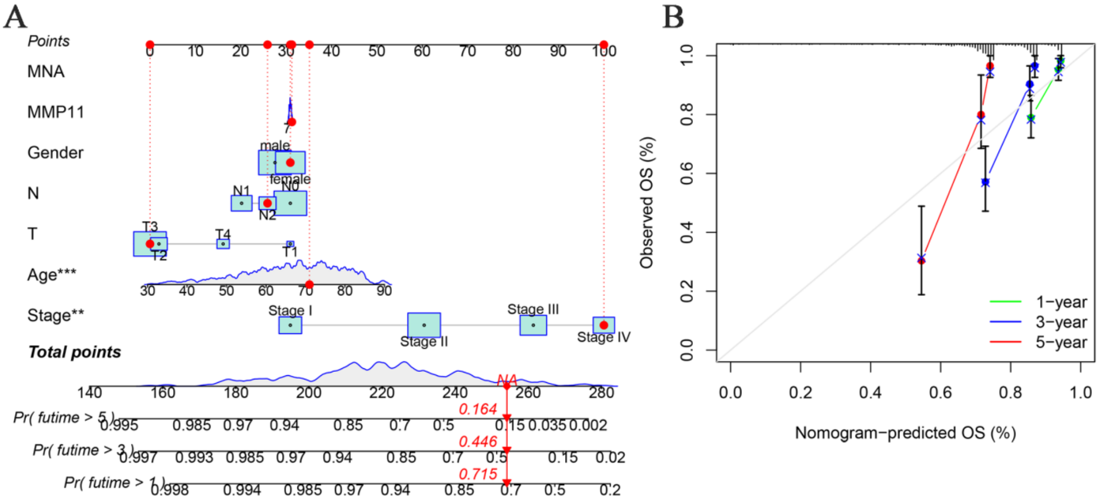

Figure S2. The construction and assessment of a prognostic nomogram. (A) A nomogram designed to predict 1-, 3-, and 5-year survival rates. (B) The calibration curves for the nomogram.

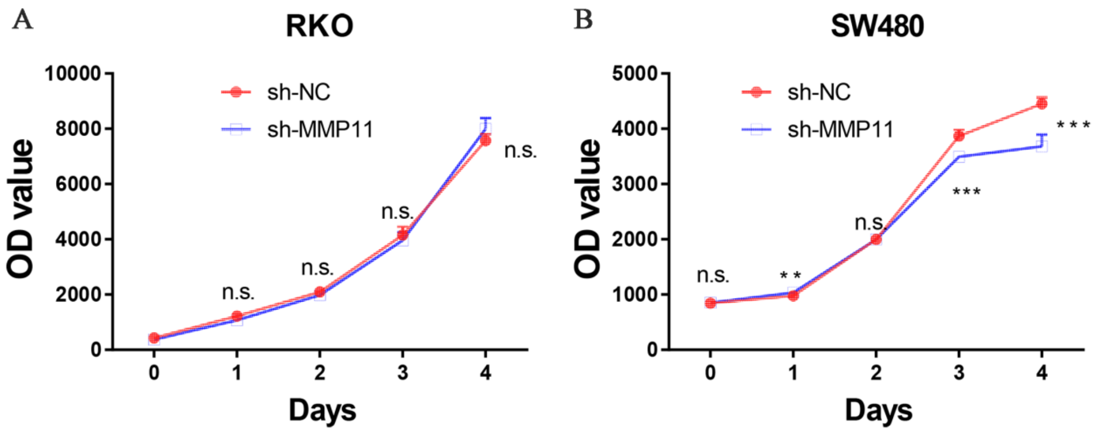

Figure S3. The progression of RKO and SW480 cells. (A-B) Optical density curves of CCK8 of RKO and SW480 cells. The experiments of cell proliferation assays were repeated three times. Data were presented as the mean ± SD and analyzed by Student's t-test. n.s. $p > 0.05$, *$p < 0.05$, **$p < 0.01$, ***$p < 0.001$, compared to the negative control (NC) group.

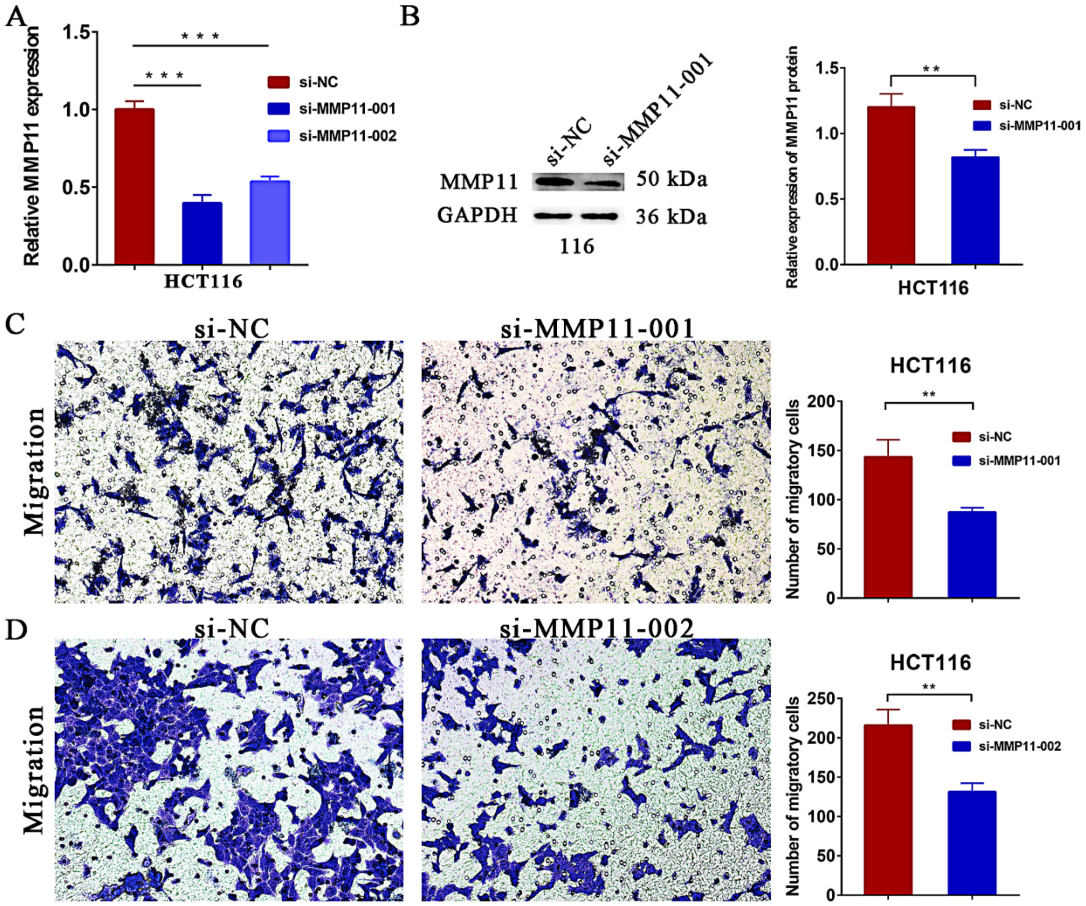

Figure S4. (A-B) qRT-PCR and western blotting revealed the expression of MMP11 in HCT116 cells transfected with different siRNA. The experiments of qRT-PCR and western blotting assays were repeated three times. (C-D) Transwell assays on HCT116 cells with different si-MMP11. The cells were stained with crystal violet. The data for si-MMP11 at the protein level were repeated three times. Data were presented as the mean ± SD and analyzed by Student' s t-test. $*p < 0.05$, $**p < 0.01$, $***p < 0.001$, compared to the negative control (NC) group.
